# Establishing a Public Involvement Network for Chronic Pain Research in the United Kingdom: Lessons Learned

**DOI:** 10.1111/hex.70373

**Published:** 2025-08-10

**Authors:** Sharon Grieve, Rosie Harrison, Carolyn Chew‐Graham, Ian Taverner, Joanne Lloyd, Noureen Shivji, Ellen Readman, Adele Higginbottom, Colin Wilkinson, Lisa Austin, Edmund Keogh, Candida McCabe

**Affiliations:** ^1^ University of the West of England Bristol UK; ^2^ Royal United Hospitals Bath NHS Foundation Trust Bath UK; ^3^ School of Medicine Keele University Newcastle Staffs UK; ^4^ University of Bath Bath UK; ^5^ Dorothy House Hospice Care Winsley Bradford‐on‐Avon UK

**Keywords:** chronic pain research, public contributor, public involvement

## Abstract

**Introduction:**

The Consortium to Research Individual, Interpersonal and Social Influences in Pain (CRIISP) is a 4‐year UK university collaboration investigating how thoughts and feelings, personal relationships and lifestyle can affect chronic pain. Patient and public involvement in research recognises that researchers' conceptions of health and illness can be enriched and sense‐checked by those of people experiencing a health condition. Published literature reports a gap in meaningful patient and public involvement in research into chronic pain, for example, during early study design. Input in this formative stage aimed to ensure the research proposed had a patient‐centred focus which may benefit study implementation.

We describe how the authors sought to address this gap and established a diverse public involvement (PI) network to support the CRIISP research.

**Methods:**

Thirty‐six adult public contributors were appointed to work alongside the research teams. Lessons learned are presented under the themes: optimising collaborative working, recruitment of public contributors, supporting public involvement throughout CRIISP and the retention of public contributors. Throughout this paper, we refer to the term ‘public involvement’ rather than ‘patient and public involvement’ in accordance with the NIHR definition which incorporates people with a range of experiences.

**Results:**

Working in partnership with our public contributors, we have embedded PI throughout a chronic pain research programme using an innovative and collaborative process.

**Conclusion:**

This model may inform others to maximise the potential of PI within their research.

**Patient or Public Contribution:**

The paper reports the collaboration between public contributors with a lived experience of chronic pain and the Consortium to Research Individual, Interpersonal and Social influences in Pain (CRIISP) researchers, by means of a large public involvement network.

## 1 Background

Chronic pain, defined as pain lasting at least 3 months, is conservatively estimated to be experienced by between one‐quarter to one‐third of the United Kingdom (UK) [1, 2, 3]. A smaller proportion experience what is referred to high impact pain [4] which limits participation in activities and social interaction [5] Despite its prevalence, a recent report describes chronic pain in England as ‘unseen, unequal and unfair’ [6]. Research indicates significant disparities in chronic pain outcomes among individuals from diverse backgrounds. In England, those of Bangladeshi, Pakistani, and Black Caribbean descent report higher levels of diagnosed chronic pain compared to other ethnic groups [7, 8]. There are also known sex/gender differences, with women generally experiencing more pain and pain‐related disability [9, 10]. Health inequalities in pain experiences are shaped by social, political, and economic factors like income, power, discrimination, and living conditions [11] with growing UK evidence that poverty‐related stressors are negatively impacting health and increasing experiences of pain [12].

To help combat some of these inequalities, including people with chronic pain, or those caring for people with chronic pain, in research can help transform our understanding of how chronic pain happens, persists and changes throughout people's lives. By embedding academic and experiential conceptions of chronic pain within the research process helps to ensure the research is meaningful to people in chronic pain. It requires shared decisions to be made throughout all phases of the research, and to ensure those with lived experience of chronic pain are placed at the centre of the research. This is the essence of meaningful patient and public involvement (PPI) in pain research.

PPI is the involvement of members of the public, patients, service users and carers working alongside researchers, to shape how the research is designed, delivered and disseminated [13]. PPI is distinct from when people take part in research studies as participants. In recent decades, PPI has been increasingly recognised and is now a well‐established, expected and essential part of the design, delivery and dissemination of all UK health and social care research [14, 15, 16, 17]. More recently there has been a focus on equality, diversity, and inclusion as a critical aspect to PPI [18] including strategies for meaningful and wider inclusion of diverse communities [19].

The value of PPI includes giving a voice to those experiencing conditions, including pain, to influence research and bring a real‐life perspective, and to influence the relevance of the research and diversity of study participants [20]. There is an international drive to involve patient and public partners in research, and although high on the research agenda, it is not consistently well implemented across countries [21, 22]. The UK has led in this field, with PPI embedded as one of the key principles of the UK policy framework for health and social care research, which benchmarks good research practice [23, 24]. Indeed, integrating PPI within research can improve the quality of research at all stages (identification and prioritisation, commissioning, design and management, implementation, dissemination and evaluation) [25].

To help guide effective PPI, the UK Standards for Public Involvement were published as a reflective tool. They describe six principles of effective PPI in research: working together, inclusive opportunities, support and learning, communications, governance and impact [26]. In practice, PPI needs to be an active and integrated process where public contributors are fully empowered, equal and participatory members of the research team. Yet, there are different types of PPI‐researcher collaborations, which range from tokenistic involvement to true coproduction activities based on how the power imbalance in decision‐making between public contributors and researchers is negotiated [27].

A key question is how well have these principles been applied to pain research? Unfortunately, a recent narrative review of the involvement of patients as partners in pain research identified gaps in meaningful PPI, for example during study design, reporting that involvement is not typical or, at least, not reported in the published literature [28]. Input in this formative stage would ensure the research has a patient‐centred focus which may benefit study delivery, dissemination and implementation. Fortunately, there has recently been a major investment in UK pain research in the form of the Advanced Pain Discovery Platform (APDP) (https://apdp.community/), a national UK pain research programme funded by UKRI and Versus Arthritis that includes PPI. This paper describes the work conducted within one of the multidisciplinary consortia, the Consortium to Research Individual, Interpersonal and Social influences in Pain (CRIISP; http://criisp.uk/) and how we sought to address this PPI gap in chronic pain research by establishing a large public involvement (PI) network to support its work.

Starting in 2021, and ending in December 2025, here we describe, critically appraise, and outline the key lessons learned, from the first 2 years of delivering the CRIISP PI network. Throughout this paper, we refer to the term ‘public involvement’ rather than ‘patient and public involvement’ in accordance with the NIHR definition which incorporates people with a range of experiences [29].

The paper was written by the members of CRIISP, who led the establishment of the public involvement network, which included public contributors.

## 2 Methods

### 2.1 Inception of CRIISP Public Involvement Network

The CRIISP research programme aimed to better understand the psychosocial mechanisms of chronic pain in adults and identify factors that might lead to pain being maintained, improved or worsened. When writing the CRIISP grant application, two people with lived experience of chronic pain were included to co‐design the research programme and embed the experience of living with, or caring for those with, chronic pain. As part of the application process, potential consortia were invited to an interview with the funders, and our interview team included one person with lived experience of chronic pain who had been involved in writing the application. The research programme comprised seven clearly defined subgroups or ‘work‐packages’, each set up with the necessary membership, resources, and activities to achieve specific objectives. Table 1 comprises a CRIISP‐specific glossary of terms.

**Table 1:** CRIISP glossary.

CRIISP term	Definition
Consortium Public Advisory Group (CPAG)	A group which provided oversight of all CRIISP public involvement work under the leadership of the CPAG Chair and Vice Chair.
Consortium Public Advisory Group Chair & Vice Chair	Public contributors who took on a leadership role, working as members of the public involvement work‐package team.
CRIISP	Consortium to Research Individual, Interpersonal and Social influences in Pain.
Public contributors	Members of the public, who have experience of chronic pain, working alongside the CRIISP researchers in a public involvement role.
Work‐packages	Subgroups within the CRIISP research programme, each focused on a specific topic.
Work‐package Development Group (WDG)	Research group comprising researchers and public contributors, focused on one of the research topics.
Work‐package Development Group co‐chair	Public contributor who took on an additional role to chair the WDG and report to the CPAG.

Public involvement was intended to be fully integrated throughout the project and, to recognise this commitment, a bespoke CRIISP public involvement (PI) work‐package, which included our two public contributor leaders, was tasked to establish and support this network, the processes and governance. Once funded, and following best practice, the first step of this study‐package was to grow a large PI network [26]. Since the focus of CRIISP was pain in adults, the PI network recruited adults rather than children or young people.

In addition to the PI work‐package, CRIISP comprised an additional six work‐packages: three addressing a single topic (individual, interpersonal, and societal factors), two cross‐cutting (large cohort datasets, digital data capture), and a final one which had an overarching theme of Integration and Translation (see supplementary information); each work‐package was led by a co‐investigator working with a team of academic researchers.

### 2.2 The CRIISP Public Involvement Work Package

To reflect the cross‐university nature of CRIISP, the PI work‐package was co‐led by teams from two UK universities, with a third supporting the delivery of public involvement. The PI work‐package roles were all part‐time across the universities, with the Administrator and two Research Fellows having the most time dedicated to the project (see supplementary information). The role of Chair and Vice Chair were the first public contributor positions to be filled and taken up by the two experts by lived experience who formed part of the application writing group.

### 2.3 Growing the PI Network

One of the first priorities of the PI work‐package team was to meet with each work‐package lead to establish their requirements, which was used to inform the formation of the PI network e.g., the number of public contributors to be appointed and what public involvement may look like to inform the research. This provided an early indication of the public involvement contribution required to fulfil the research aims; however, this was constantly reviewed as the research progressed.

**Figure 1:**
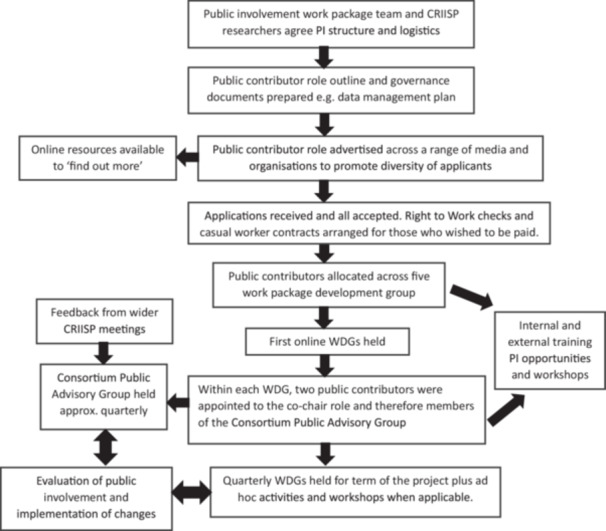
Steps to establishing the PI network.

It was important for the PI work‐package team to understand the complex structure of CRIISP and identify how PI could be fully embedded within each work‐package to enable the project to meet the research aims. PI spanned 4 years and so incorporating strategies to assure retention of the appointed public contributors was a key priority. Figure 1 depicts a flow diagram outlining the steps taken by members of the PI work package to establish the PI network.

### 2.4 Recruitment of Public Contributors

For the public involvement within CRIISP, ‘public’ was defined as people living with chronic pain or those caring for people with chronic pain. Involving these groups in a meaningful way was central to CRIISP from the outset to ensure the research reflected and was relevant to the lived experience of chronic pain. Influenced by UK Standards, the aim of the PI work‐package team was to promote mutually respectful, strengthening and productive working relationships [24].

The recruitment of public contributors to CRIISP commenced in March 2022 and was initially completed in September 2022. Due to the expected attrition in a long‐term project, there was a further recruitment drive in April 2024. Before recruitment, role descriptions, adverts and application materials were co‐designed by the PI work‐package team. University employment guidance was adhered to throughout.

An advertisement invited applications from adults in the UK who were willing to share their experience of living with chronic pain or caring for someone with chronic pain. The views of unpaid carers are often overlooked but it was considered important to include this different perspective and/or enable them to advocate for the person in pain [30]. Previous experience of PI was not essential and bespoke training was offered to tailor specifically to the CRIISP programme of research. PI is often reported as failing to be inclusive of under‐served communities [31] so a proactive and inclusive recruitment strategy sought to optimise diversity in terms of sex/gender, age, ethnicity, socioeconomic status and prior experience of PI work. An eye‐catching advertisement using accessible language was shared with equality organisations, women's groups, community groups as well as pain charities, local networks and via social media (see supplementary information). A bespoke CRIISP website was developed for the duration of the programme and dedicated PI pages were incorporated (https://criisp.uk/ppi-2/). Interested applicants were directed to this for more information on the roles and how to apply. A ‘Find out More’ video was produced by the PI work‐package to improve accessibility and engagement (https://criisp.uk/get-involved/). The application form included an invitation to share any relevant skills and experience and provide a short statement of why they were interested in the public contributor role.

Applications were reviewed by members of the CRIISP public involvement team. A decision was made to accept all applicants to ensure public contributors had a range of backgrounds and expertise.

### 2.5 Public Involvement Roles Within CRIISP

**Figure 2:**
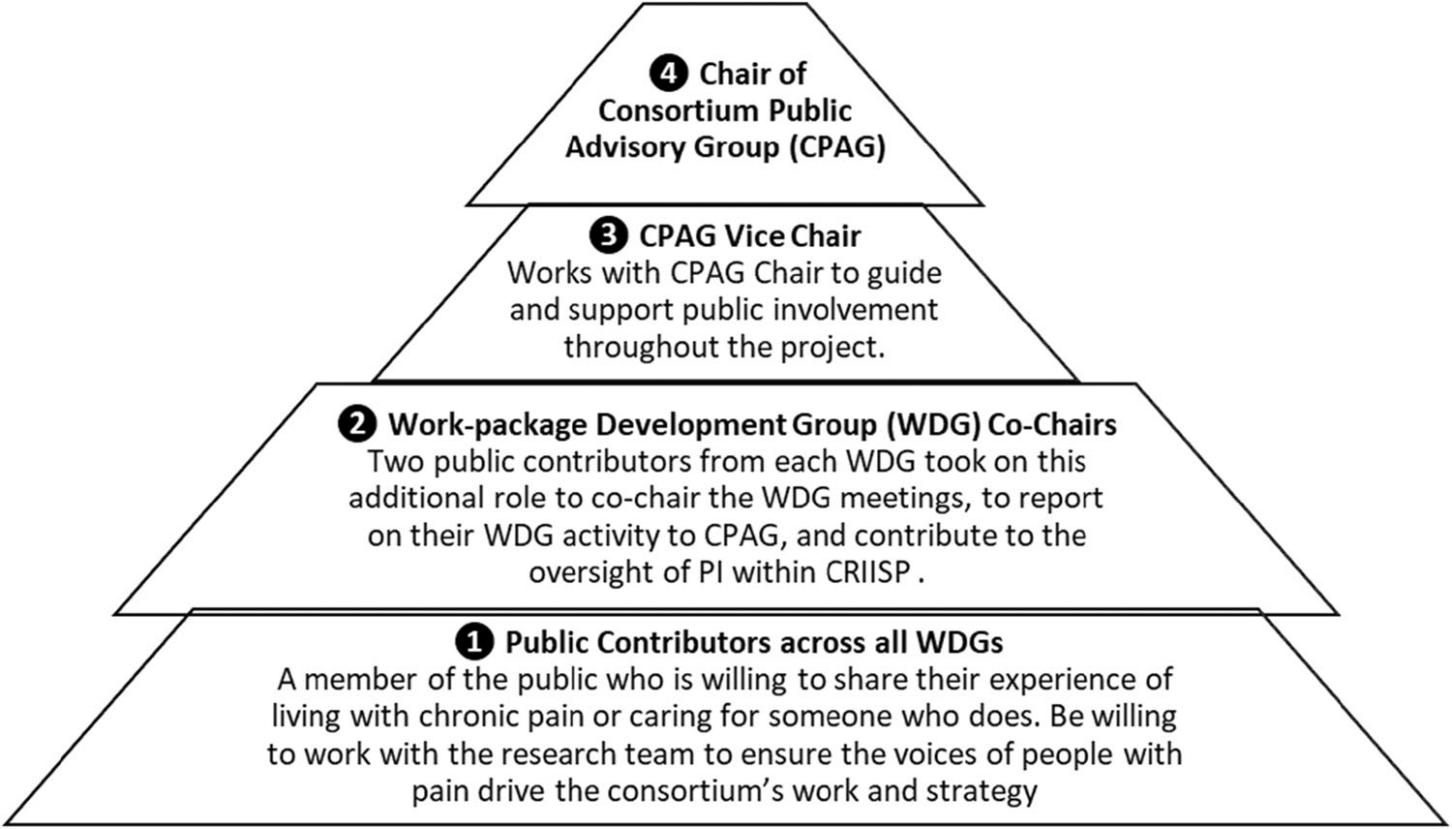
CRIISP PI roles.

A tiered approach to PI was devised, with four distinct roles for public contributors (Figure 2).

1. Public contributors across all Work‐package Development Groups
  A Work‐package Development Group (WDG) was established for each work‐package (or research group). The aim was to ensure each work‐package had a small group of public contributors (Figure 2, ❶) who could help develop its work, and work alongside the researchers in relationship‐driven, rather than transactional manner. Meeting online on a quarterly basis, each WDG: (1) worked together with researchers to decide how to conduct the research, and ensured the research reflected both what it was like to live with chronic pain as well as academic conceptions, (2) acted as critical colleagues to the researchers, asking questions, sharing in decisions and providing constructive feedback, (3) provided feedback on study design to ensure it would be acceptable to people living with chronic pain, and (4) ensured participant‐facing study documentation are communicated in plain language.
  Each public contributor was allocated to a WDG based on their interests and experience, and their sex/gender, age and ethnicity, to ensure diversity across all WDGs. The public contributors were introduced to their WDG research team and public contributor colleagues via an online induction meeting which included an outline of the scope of the WDG and background to the work.
  The quarterly WDGs ensured the researchers maintained a focus on the lived experience, with the additional benefit of promoting the regular engagement and commitment of public contributors.
2. Work‐package Development Group Co‐chairs
  Within each WDG, two public contributors were appointed to WDG co‐chair roles (Figure 2, ❷). Expressions of interest were invited from members of each WDG and/or direct approaches made by the CPAG Chair and Vice Chair to identify potential candidates.

### 2.6 3 & 4. Consortium Public Advisory Group (CPAG) Chair and Vice Chair

The Consortium Public Advisory Group (CPAG) was created to oversee, guide, and manage the CRIISP public involvement work under the leadership of the CPAG Chair and Vice Chair (Figure 2, ❸❹), and to offer CRIISP researchers an opportunity to discuss cross‐cutting research issues with public contributors who brought knowledge of each work package. CPAG membership also included the co‐chairs of each WDG, the CRIISP Principal Investigator and project manager, and the PI work‐package Co‐Leads

The Chair and Vice Chair had four key roles. They were: (1) public contributor members of the PI work‐package team, (2) members of the CRIISP governance board, (3) chair of meetings of the Consortium Public Advisory Group (CPAG), which provided public involvement oversight and governance and (4) acted as critical friends and mentors to the co‐chairs of the WDGs. Between them, they attended all research meetings that included public contributors and some meetings that did not. Having public contributors in these leadership roles ensured the entire CRIISP structure had public involvement embedded.

The CPAG also played a role in ensuring shared research decision making between public and academic contributors. CPAG members sought opportunities for work‐packages to collaborate to maximise the impact of CRIISP's work. The CPAG met quarterly or at critical points in the CRIISP work. As the CRIISP research progressed, CPAG was utilised as a platform to explore new ways of working with public contributors and to develop ideas for research dissemination and supported the development of future grant applications.

### 2.7 Infrastructure of the Work‐Package Development Groups

**Figure 3:**
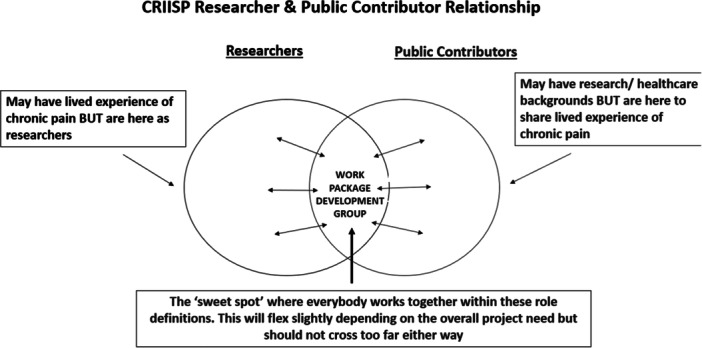
CRIISP researcher and public contributor relationship [32].

Each WDG comprised the work package research team, between 5 and 7 appointed public contributors (two of whom were appointed co‐chairs), the CPAG Chair or Vice Chair, and one of the PI work‐package Research Fellows. Delegating one Research Fellow to each WDG minimised duplication of workload (e.g. co‐ordinating meetings) and enabled a productive working relationship to be built with the researchers and public contributors. The Research Fellow and CPAG Chair/Vice Chair provided continuity within the WDG and worked with the research team before, during, and after the WDG meetings, to support the public and academic contributors to share decisions about the research in a productive manner (Figure 3).

Feedback from the public contributor co‐chairs after the initial series of WDG meetings prompted the implementation of a 30‐min meeting a couple of weeks before every WDG. This was held with the CPAG Chair, WDG lead researcher, Research Fellow Link and co‐chairs. It provided: (1) an opportunity for the co‐chairs and academic contributors to prepare for the upcoming WDG meeting, (2) to ensure the agenda and time allotted were appropriate for the desired outcomes, and (3) to review any pre‐WDG activity and planned WDG meeting content to ensure it was accessible and easily understood by public contributors. It was also an opportunity to agree how to frame challenging or difficult content, if applicable.

### 2.8 Communication Between Public Contributors and Researchers

The PI work‐package team selected MS Teams® as an effective and secure method of convening online meetings and sharing and collaborating on documents. It was easily and freely accessible via a publicly available platform and had a positive track record with Consortium staff when used during the pandemic. Cross‐university working added a layer of complexity as working practices differed across organisations. Work package‐specific Teams® Channels were set up and access was granted according to the individual's role. In response to demand, the PI work‐package administrator provided additional resources such as step‐by step user guides, one‐to one technology and MS Teams support®. Specialist equipment, such as a web cam, was provided to applicants who met pre‐defined criteria.

### 2.9 Supporting Our Public Contributors

An early priority for the PI work‐package was to establish the payment process for our public contributors. UK guidance informed administrative decisions and facilitated discussions with the university to incorporate the CRIISP pay scale [33]. Recent guidance suggests rates of pay but these are a benchmark only [34]. A range of payment methods are used across the UK [34] and this lack of consistency made it more challenging to unpick. ‘Casual worker’ contracts were required for the public contributors according to university policy and they were paid for their time according to the costs included in the CRIISP grant. After completion of a university registration form and a UK government authorisation of the ‘Right to Work’ in the UK, a casual worker agreement was issued to each public contributor which set out the terms of the arrangement. The exception was those who wished to undertake the role on a voluntary basis.

The public contributor Chair and Vice Chair were employed on a part‐time basis by the lead university.

Resources were developed by the PI work‐package to support the public contributors in their work with CRIISP (see supplementary information). The resources included a guidance document for researchers which outlined the steps to take to support public contributors' wellbeing if any issues were reported. In addition, public contributors had access to a similar document which directs them to a university confidential staff helpline. The public contributors were encouraged to contact the work‐package Research Fellow if they had any unmet support needs, and an email was circulated annually to remind public contributors to ‘reach out’ for support as required. Resources were informed by the principle that those involved in research should be treated fairly, with dignity and respect, reasonable steps need to be in place to mitigate risks for exploitation, abuse and harm within research activities [35].

In addition, public contributor only ‘de‐brief’ session was introduced at the end of each WDG meeting, led by the Chair or Vice Chair of CPAG. The purpose was to gather informal feedback on each session. Public contributors received quarterly newsletters produced by the PI work‐package team which included: CRIISP research and staffing updates, training and involvement opportunities and required administrative actions. Public contributors and researchers were invited to contribute blogs to the CRIISP website and articles to the newsletter, to share their PI experience and learning.

### 2.10 Evaluation of Public Involvement

Three separate online survey evaluations were conducted up to September 2024: (1) an evaluation of the public contributor's experience of the WDG application process, (2) an evaluation of the researchers' experience of public involvement (3) an evaluation of the public contributors' experience of being involved in their WDG and the support they have received. The findings informed the results reported below.

### 2.11 Dissemination

**Figure 4:**
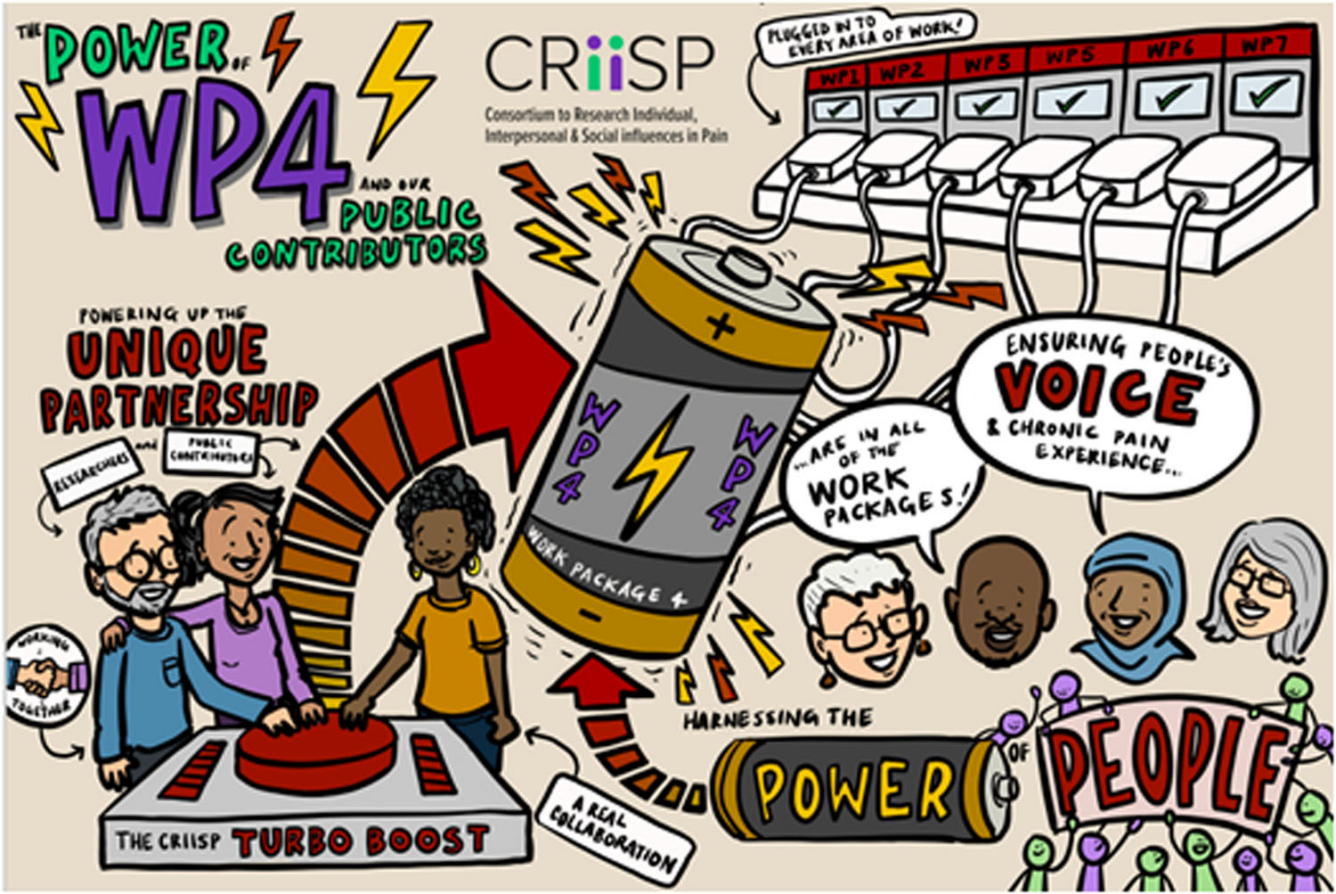
Public involvement illustration by Tom Bailey.

Public contributors were integral to the dissemination of the CRIISP research, including advising researchers on avenues for sharing findings. Dissemination activities up to March 2025 included: contributing to a key paper proposing a framework for the establishment, maintenance, and adaptation of high and low impact chronic pain [4], writing blogs to share their experience of CRIISP (https://criisp.uk/blog-2/), a poster presentation at an international conference [36], coproduction of CRIISP events with the PI work‐package team, co‐authored abstracts for national and international conferences, and presenting the CRIISP output as conference speakers (https://criisp.uk/category/public-contributors/). An illustrator was commissioned to capture the essence of CRIISP public involvement in visual form; an example can be found in Figure 4. These images will be used for future dissemination purposes. Dissemination is ongoing at time of writing.

### 2.12 Results Recruitment to the Public Involvement Network

Twenty‐eight public contributors were recruited (March‐ September 2022) and full public involvement activities commenced in September 2022. Eight public contributors have since withdrawn from involvement due to life events and availability (updated to March 2025). This is to be expected for a research programme over several years duration. To maintain sufficient involvement, eight additional public contributors were recruited to several WDGs in March—June 2024. They were approached with consent from a list compiled of those expressing interest in the role after the first tranche of recruitment was completed. Table 2 describes the characteristics of all CRIISP public contributors until September 2024. We were successful in attracting representation from younger adults (*n* = 5) and male public contributors (*n* = 7) which is not always the case in public involvement. Although underrepresented overall, male public contributors were well represented in the ten co‐chair roles (*n* = 5). The co‐chair roles were offered to all public contributors; however, we recruited on experience and ‘fit’ which resulted in an equitable gender split.

**Table 2:** Characteristics of public contributors.

Characteristics of CRIISP public contributors	
Gender	29 F 7 M
Age	18 ≤ 34 = 5 35 ≤ 64 = 26 > 65 = 4 Undisclosed = 1
Ethnicity	White British = 26 White Eastern European = 3 Mixed ethnic = 3 Black = 2 Asian = 1 Undisclosed = 1
Location	Scotland = 3 Wales = 2 England – North = 9 England – Mid = 5 England – South = 17
Person with chronic pain/Carer of person with chronic pain	Person with chronic pain = 31 Carer = 2 Both = 3
Duration of chronic pain	3 months ≤ 2 years = 5 3–5 years = 5 6–10 years = 6 > 10 years = 20
PI Contribution	33 = paid 3 = voluntary at their request
Withdrawn	7F 1M

### 2.13 Reflection and Critical Perspective

Truly embedding meaningful public involvement throughout the life of CRIISP had been the key objective from the outset; from inception with two public contributors helping to develop the planned work within the application, to public contributors embedded within each work package and the governance structures, to public contributors being involved in dissemination activities.

The duration and complexity of the research meant our structures looked quite different to the usual ‘public involvement’ often found in health research; where the PPI informs the funding application and is a consultative approach thereon [37]. There was a huge benefit in working with the public contributor Chair and Vice Chair as members of the Public Involvement Work Package. They constantly advocated for public involvement and ensured it was included at every stage of CRIISP, as opposed to public contributors only being invited to be involved when the researchers may believe it was required. As a result of our innovative approach, PI was integrated within every CRIISP work package and public contributors worked in true partnership with researchers to inform the research. It is acknowledged however, that many research studies do not demand (or need) this level of complexity and Table 3 provides guidance on adaptations for settings which are not multi‐centre collaborations, or where funding is limited.

**Table 3:** Challenges and lessons learned.

Issue	Challenges encountered	How we resolved it	Lessons learned
**Optimising collaborative working**			
Communicating with public contributors	Initially public contributors were unsure of who to contact for what purpose and were frustrated when they did not receive a same day response to queries. This was compounded by the research fellows and administrator working across institutions on part‐time contracts.	Allocating a Research Fellow to each WDG as a named point of contact. Clarity on working hours via email signature. Regular meetings between research fellows and administrator (who work in different institutions) to resolve queries from public contributors. Clear communication with public contributors regarding working hours and availability of staff to manage expectations.	Maintaining engagement with public contributors requires regular and responsive communication. Public contributors appreciate knowing who to contact, during which hours, for what purpose, and an alternative contact for when the primary contact is unavailable.
Using MS Teams®	The functionality of MS Teams® was not always clear for public contributors or researchers. There were also some initial difficulties in accessing Teams across institutions.	We created guides for public contributors on how to access and use their MS Teams® channel. We also provided individual tutorials to resolve specific issues.	Using MS Teams® channels was an effective and secure way of communicating with public contributors, providing an opportunity to work collaboratively and in real time. However, to use it effectively many public contributors and researchers require initial and ongoing technical support.
Processes risked becoming bureaucratic and time‐consuming	Working cross‐institutionally within the PI work‐package and across CRIISP necessitated the navigation of many procedures and policies that were sometimes contradictory. We created documents to aid PI in CRIISP but this could be time‐consuming, e.g., when documents developed by the PI work‐package needed to be adapted following feedback from the wider CRIISP community.	Monthly PI work‐package meetings were used to collectively make decisions on procedures and documents. Interim work was led by smaller groups from the PI work‐package to facilitate timely delivery.	Clear processes and documentation are key to working within a large project, but creating these can be time consuming. It was crucial to have ways of making collaborative decisions in a timely way.
**Recruitment of public contributors**			
Recruitment processes	We encountered issues with payments as our public contributors were having regular interactions not one‐off activities. Navigating the university procedures whilst adhering to the NIHR guidance was often difficult. Additionally, some public contributors chose not to be paid due to the adverse effect this would have on UK Government benefits, pensions or tax contributions. This was our UK‐specific experience and raised issues of equality and fairness that these people were not compensated for their time. Public contributors were also required to have a ‘right to work in the UK’ check and the new online platform proved difficult for new public contributors to access.	We liaised with university teams at a higher level to ensure we were adhering to university and NIHR guidance regarding payments. As PI was not a one‐off event university policy stated we were not allowed to provide public contributors with alternative methods of compensation (e.g. vouchers). We liaised with the university human resources team to resolve any issues with the ‘right to work’ checks.	Payment of public contributors may differ across countries, cultures and organisations. In the UK, University processes regarding PI payment and recruitment can be challenging to navigate. It would be useful to understand how specific policies will interact with how public contributors are embedded within the project to prevent or foresee future difficulties. Administration staff were key to managing these issues and liaising with other university departments.
Diversity of public contributors	We struggled to recruit an ethnically diverse public contributor group despite advertising through national public networks. Advertising the opportunity was hampered at times as charitable and community organisations sometimes requested payment or required completion of lengthy forms.	We held focus group discussions with existing public contributors to brainstorm ideas on how to reach a more diverse and underserved group of public contributors. We joined regional networks with a focus on increasing diversity in research involvement and participation.	Advertise well in advance and utilise networks to identify community groups. Engage the support of people within those communities to raise awareness of the public involvement opportunities on the behalf of the research team. Take time to establish a meaningful working relationship with community leaders to establish trust. This ideally needs to be in place before the project begins.
**Supporting public involvement throughout CRIISP**			
Enabling public contributors to make a reasonable contribution to WDGs and supplementary activities	Many research terms were new to the public contributors. Research concepts may have contested meanings and are shaped by disciplinary conventions and tradition, and using the internet or other sources may reveal differing and competing definitions when searching for these terms.	A glossary was produced specific to each WDG plus an additional public‐facing glossary available via the CRIISP website developed in conjunction with CRIISP researchers and public contributors (https://criisp.uk/glossary-for-public-contributors/). Researchers were encouraged to prepare presentation slides in advance so they could be reviewed before meetings by all members. Following early feedback from public contributors, all written materials are provided in a dyslexia‐friendly font.	While not intended to contain all possible research terms, glossaries are a useful reference point for how researchers are using key concepts. Slides need to contain pertinent information only and be designed with access needs in mind. On reflection, the CRIISP‐specific terminology added an additional layer of complexity to the research language. Involvement may have been facilitated with more lay terminology.
The content of the WDG meetings may be emotionally challenging	People attend the meetings bringing different life experiences and have, and/or care for someone with, health conditions so may not always be feeling in an optimum health state or at their most resilient.	A ‘check‐in’ was used at the start of every meeting. A short de‐brief was led by the CPAG Chair or Vice Chair after each WDG meeting to give the public contributors the opportunity to feedback on the WDG experience, for example, what worked well and where changes to the meeting structure are needed. The WDG meeting slides can be reviewed before the meeting so the members can prepare for participation. They can then decide in advance how much of their lived experience they wish to share.	Processes should be in place to signpost members if they become distressed during a WDG or express any unmet support needs. A check‐in enables everyone to ease into the proceedings and share any information which may be relevant to their participation.
**Retention of public contributors**			
Public contributor turnover	As this was a 4‐year programme of research, public contributor turnover was inevitable. Public contributors had family or health situations which required their temporary absence or withdrawal, often at short notice. This led to challenges in covering and replacement roles.	We took an individualised approach in managing the involvement of public contributors. This included negotiating temporary breaks from their role or accepting their resignation; in addition to ensuring their role was adequately covered in their absence.	Working with people living with long‐term health conditions requires an understanding of their experiences and challenges. Public contributors need to know who to contact if assistance or adaptations to their role are required.
Researchers' understanding of PPI	New researchers joining CRIISP often had limited experience of PPI or were used to a more ‘show and tell’ approach. Additionally, whilst experienced researchers had previous PI experience there were differences in how PI was understood from institutional and disciplinary norms. This caused issues with their interactions with public contributors.	Having a named Research Fellow facilitating the PI in each work package was crucial in managing expectations and articulating how CRIISP was designed to operate. The PI work‐package meeting has WDGs as a standing item to discuss and negotiate any difficulties. The Consortium Public Advisory Group was used by the co‐chairs to raise any problems encountered from the public contributor perspective. New researchers who were involved in the WDG meetings received an induction froma PI work‐package team member.	Awareness that not all researchers or institutions perceive PI in the same way, so clarity of vision and articulation of how PI is embedded within the research is crucial. Leadership from senior researchers modelling good PI practice for less experienced colleagues is also essential. Regular team meetings provided the opportunity to manage any problems encountered, including the ability to escalate problems to a higher level.
Maintaining the engagement and commitment of public contributors	The pace of research can vary throughout the programme and was dependent on the stage of the research process and methodology.	In response to feedback from the research teams, a more flexible approach to WDG meeting timings was adopted from Year 2 of CRIISP with meetings held every 3–4 months, with an interim ‘shorter’ meeting if required. This enabled full, proper and timely involvement that was responsive to research need. Quarterly newsletters were produced to provide public contributors with a wider view of the CRIISP research than their specific work package. A ‘Reconnect’ workshop was held to strengthen the public involvement sense of community and shared goals, to increase understanding of the role of the WDGs and their impact, to refresh motivation and commitment to the CRIISP work.	Innovative ways of building the public involvement community should be developed, with input from the public contributors themselves. Public contributors are invited to write items for the newsletter. The'Reconnect' workshop was co‐produced by public contributors. In Year 3–4, cross‐work package working offers an opportunity for the public contributors to become involved in the wider CRIISP work. It also enables the researchers to utilise wider diversity and experience of the public contributors.

From the outset, our approach to public involvement was one of equal collaboration between public contributors and researchers. This form of collaboration can be regarded as ‘co‐operation’ [27] where public contributors and researchers work together to decide priorities, but researchers led the process and thus it sat between traditional types of public involvement and co‐production. Within this type of collaboration, a power imbalance may exist between the public and the researchers; in terms of decision making due to the needs of research to be rigorous, drawing on existing research, and plans being viable within the temporal and financial constraints of the project. To aid this equitable collaboration between researchers and public contributors, clearly defined roles were created, with the emphasis on public contributors providing their expertise from living with chronic pain, and researchers providing their expertise in research design and implementation.

Since we established our PI network in 2022, inclusion at all stages of research is now a UK national priority [38, 39] and a key theme in the NIHR Research Inclusion Strategy [40]. The literature describes many challenges in attracting people from underrepresented groups to public involvement, for example, a fear of not being treated with dignity based on previous healthcare experience [31]. Despite a targeted strategy, we experienced challenges in recruiting a diverse group of public contributors. Two UK surveys which found those involved in research are most likely to be over 50 years, female and white [41, 42] closely reflecting our experience. Some success in increasing diversity has been reported by establishing groups which encourage conversations between community members and health researchers [43] however, the timeframe in which we were required to set up the network meant this was not feasible and it would have been beneficial to have established these connections before the project commencing. Reaching out to community groups to promote the opportunity on our behalf had limited success which may be due to the lack of visibility of the research team to establish respect and trust. We did, however, have diversity of public contributors across all topic areas at the outset, in both gender and ethnicity, even though we would like to have increased this representation. In future, linking in with community groups at an earlier stage would be beneficial.

Table 3 provides further critical reflection on our methods and reports the key lessons learned from the experiences of the PI work‐package team, and public contributor and researcher evaluations. These are reported across several themes and can be adapted for public involvement across a range of settings.

**Remaining Project Time** CRIISP will close in December 2025. Between the time of writing and the project end we will continue to evolve and adapt our approach as the work progresses and disseminate lessons learned for PPI in large research programmes.

## 3 Conclusion

We have demonstrated how public involvement can be embedded throughout a research programme using innovative and collaborative processes. Working in partnership with our public contributors, we believe we have ensured their voices are heard and our research has been significantly enriched by their contributions. We anticipate that by presenting our lessons learned and best practice, this model may inform others to maximise the potential of PI within their research.

## Author Contributions

**Sharon Grieve:** conceptualisation, project administration, writing – original draft, writing – review and editing. **Rosie Harrison:** conceptualization, writing – original draft, writing – review and editing. **Carolyn Chew‐Graham:** conceptualization, writing – review and editing. **Ian Taverner:** writing – review and editing. **Joanne Lloyd:** writing – review and editing. **Noureen Shivji:** writing – review and editing. **Ellen Readman:** writing – review and editing. **Adele Higginbottom:** writing – review and editing. **Colin Wilkinson:** conceptualization, writing – review and editing. **Lisa Austin:** writing – review and editing. **Edmund Keogh:** conceptualization, writing – review and editing. **Candida McCabe:** conceptualization, writing – review and editing.

## Ethics Statement

The authors have nothing to report.

## Conflicts of Interest

Edmund Keogh reports unrelated consultancy services via the University of Bath to Reckitt Benckiser Health Limited. Edmund Keogh has also received additional research grant funding from Versus Arthritis and UKRI (Medical Research Council, Biotechnology and Biological Sciences Research Council, Economic and Social Research Council). Carolyn Chew‐Graham is part funded by West Midlands Applied Research Collaboration.

## Supporting information

Supp material 2JAN2025.

## Data Availability

Data sharing not applicable to this article as no datasets were generated or analysed during the current study. Data sharing is not applicable to this article as no new data were created or analysed when establishing the public involvement network.
