# Supplementary material for: Establishing a Public Involvement Network for Chronic Pain Research in the United Kingdom: Lessons Learned
Source: Health Expect. 2025 Aug 10;28(4):e70373. doi: 10.1111/hex.70373 (PMC12336369; doi:10.1111/hex.70373)
Supplement: Supplementary file 1 — Supp material 2JAN2025. [file HEX-28-e70373-s001.docx]

**Su****pplementary material**

The seven CRIISP work packages.

1. Roles within the Public Involvement Work Package
2. Working Together document
3. Guidance for the involvement of public contributors in the Work package Delivery Groups (WDGs)
4. WDG role outline
5. Advert
6. Co-Chair WDG feedback form
7. **The seven CRIISP work packages.**


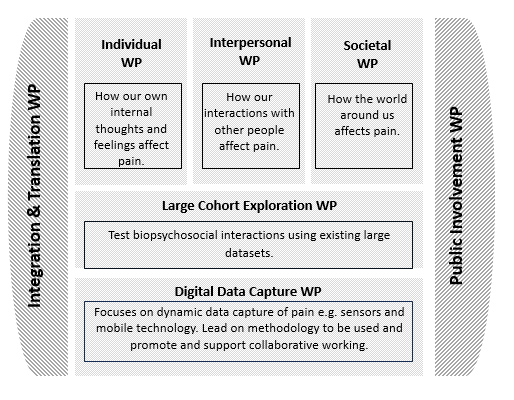


1. **Roles within the Public Involvement Work Package**

**
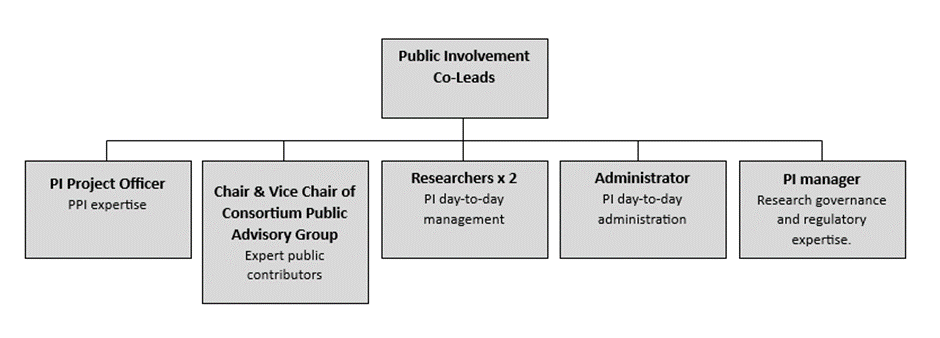
**

1. **Working Together document**

**
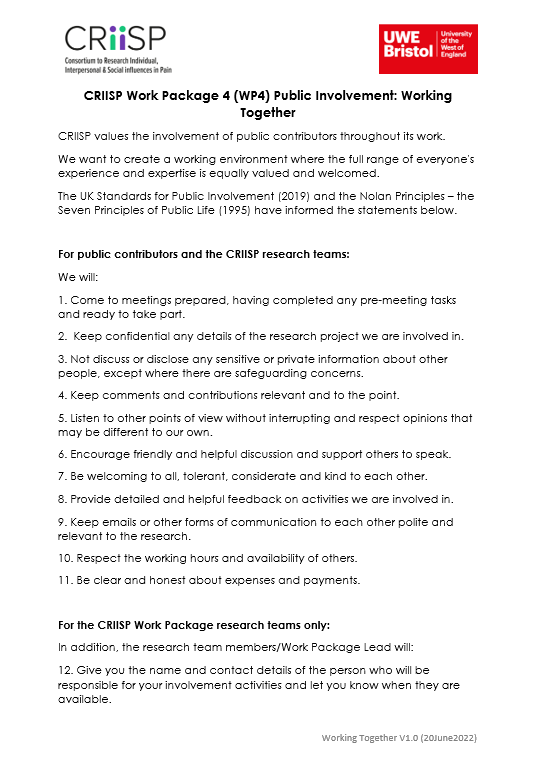
**

**
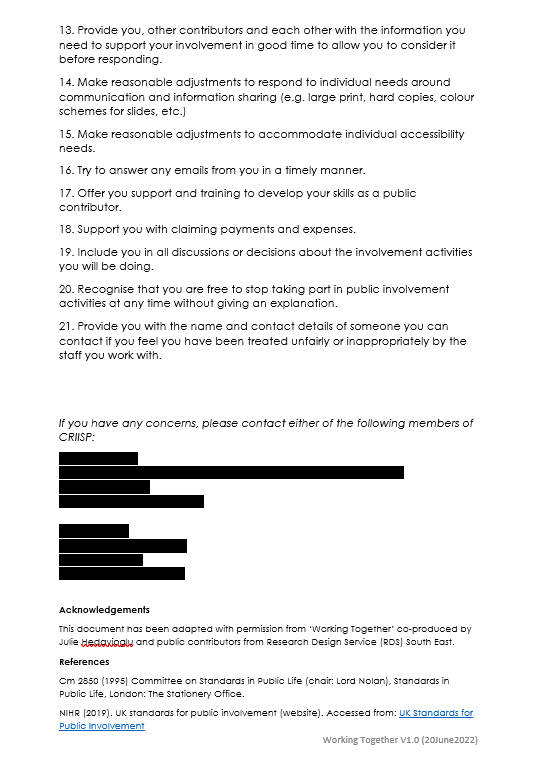
**

**D. Guidance for the involvement of public contributors in the Work package Delivery Groups (WDGs)**

**
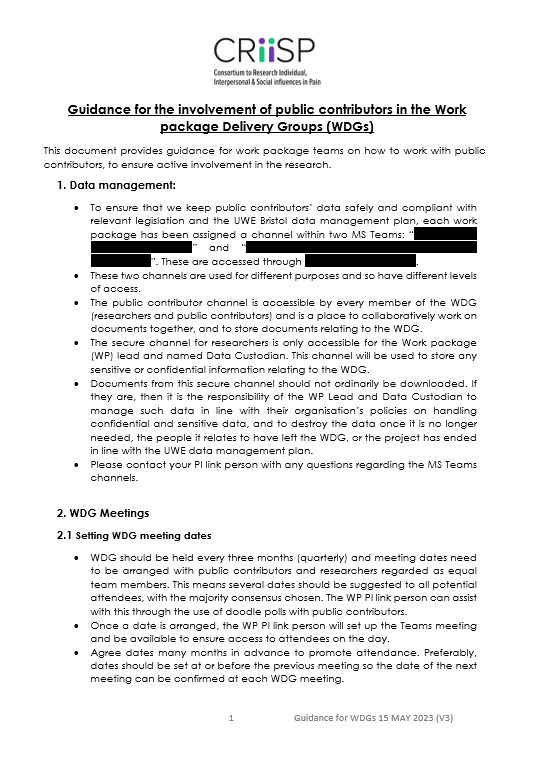
**

**
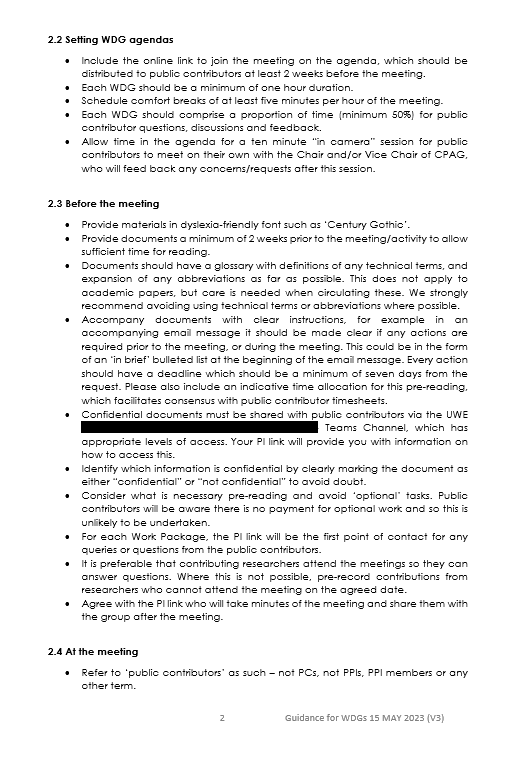
**

**
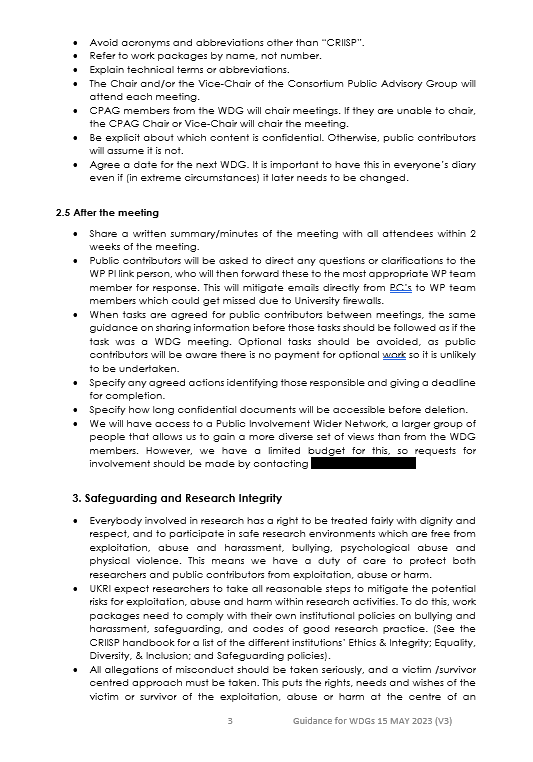
**

**
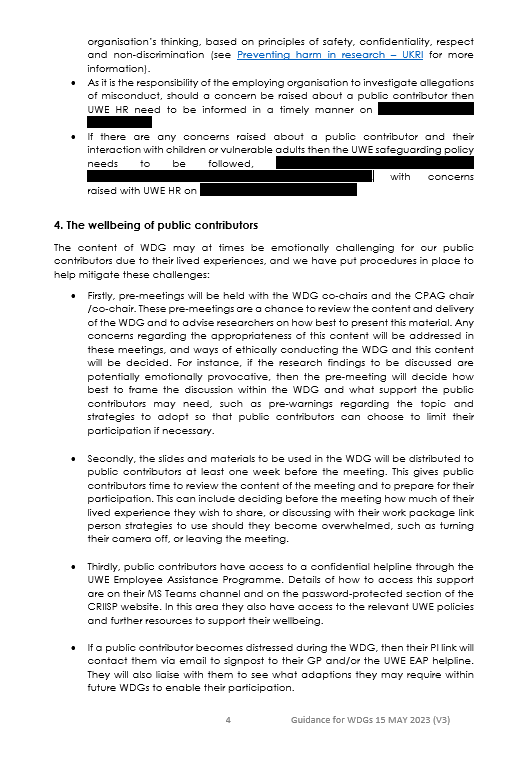
**

**
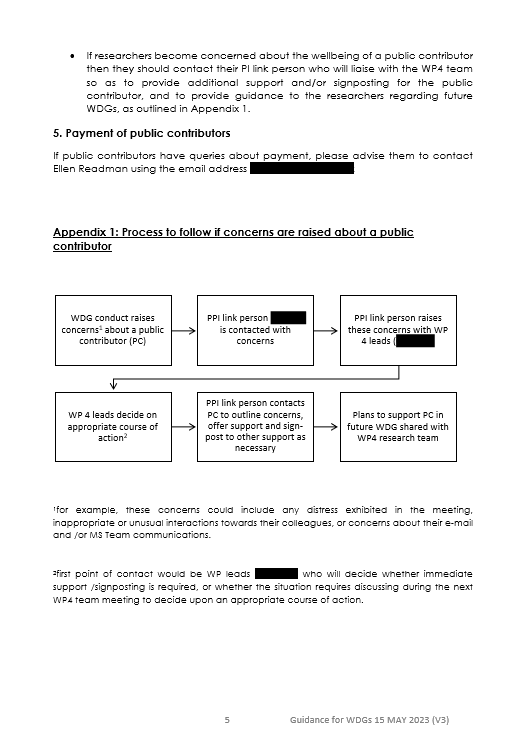
**

**E. WDG role outline**

**
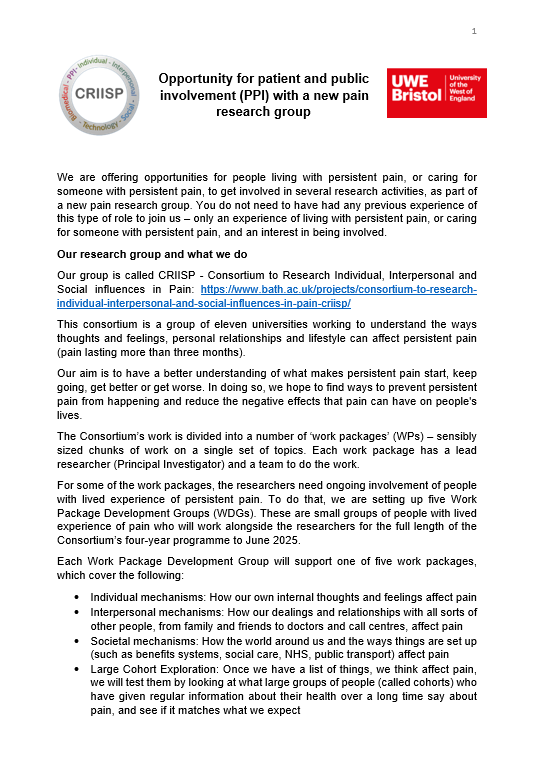
**

**
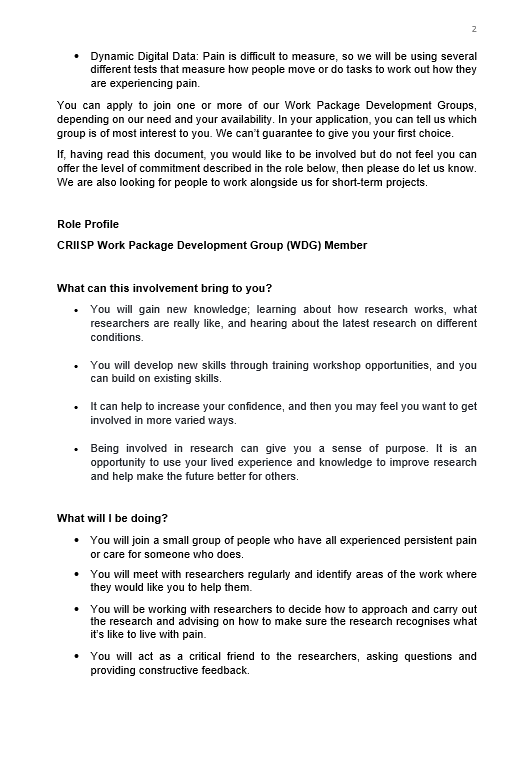
**

**
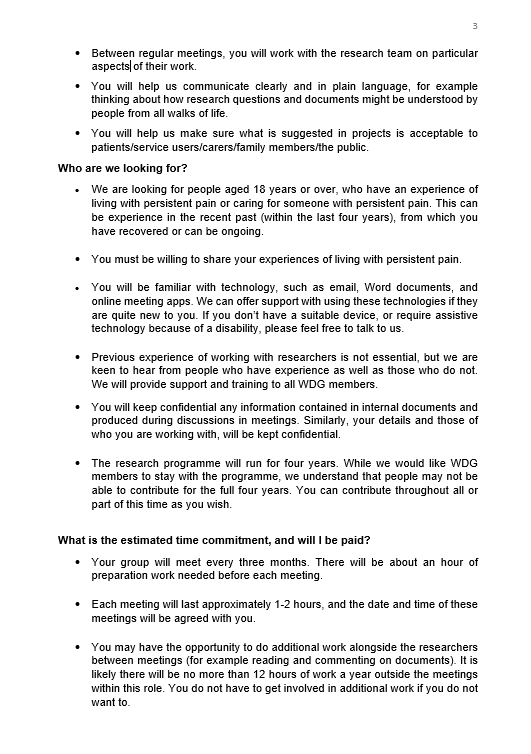
**

**
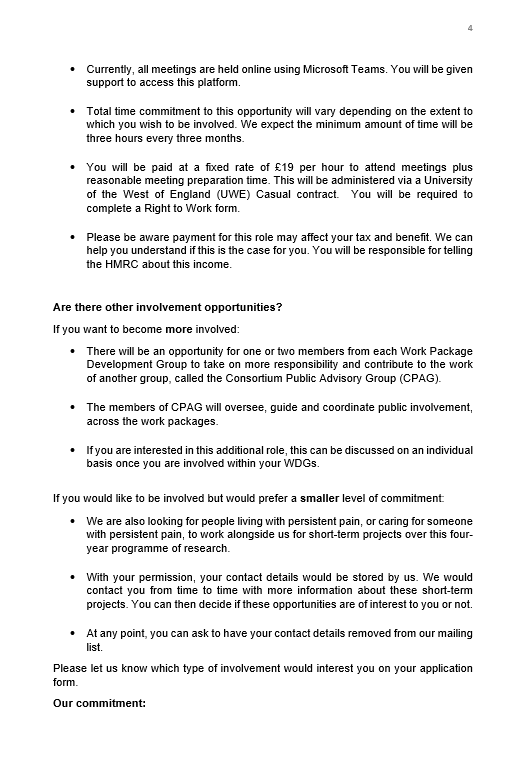
**

**
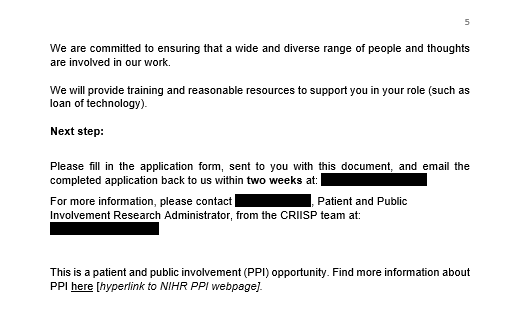
**

**F. Advert**

**
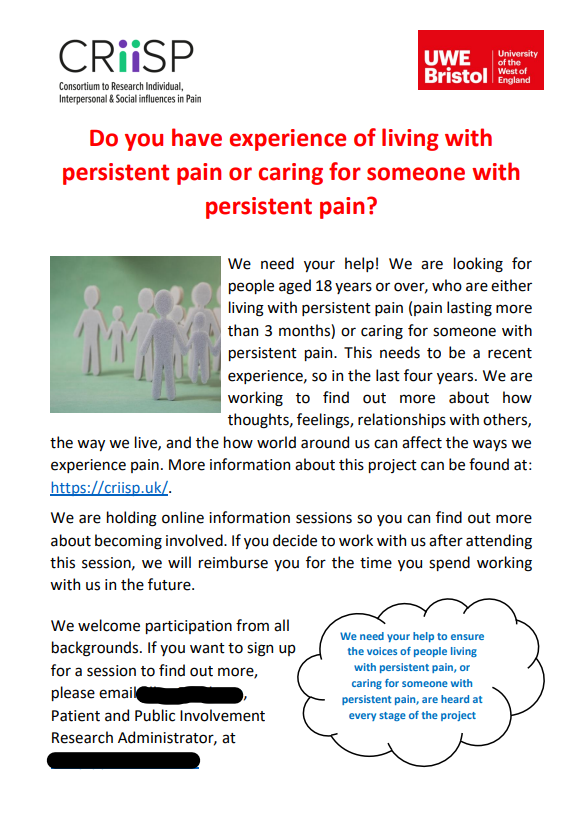
**

**G. Co-Chair WDG feedback form**

**
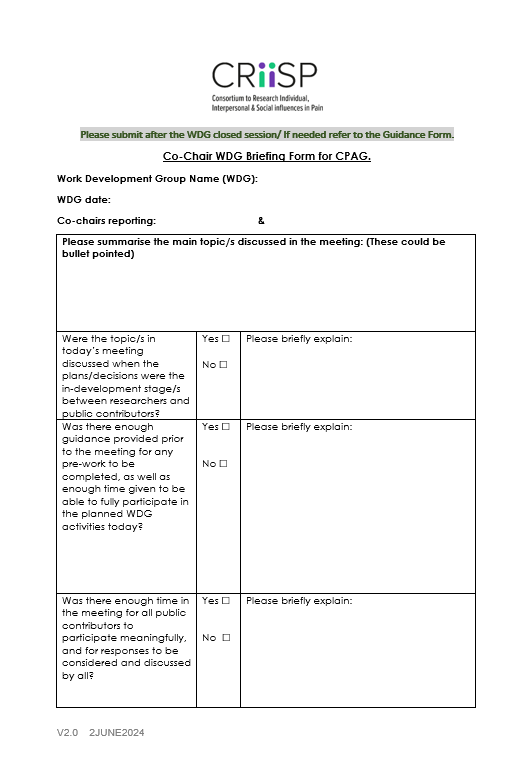
**

**
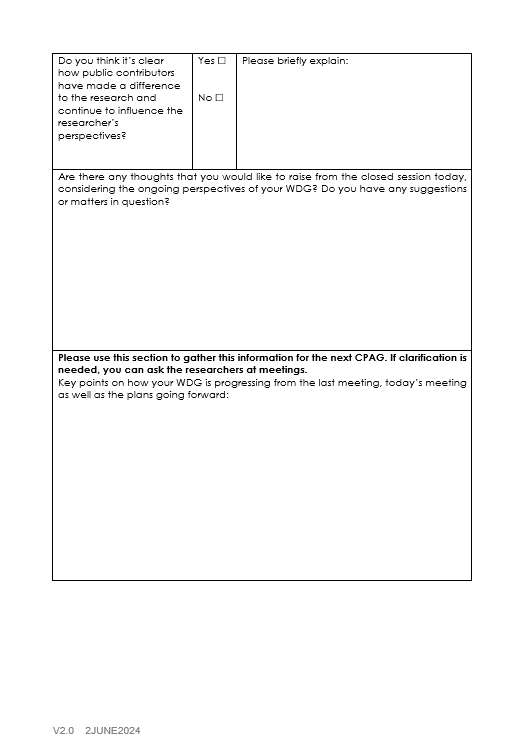
**
